# Methodology and Characterization of a 3D Bone Organoid Model Derived from Murine Cells

**DOI:** 10.3390/ijms25084225

**Published:** 2024-04-11

**Authors:** Jaymes Fuller, Katherine Sares Lefferts, Pooja Shah, Jessica A. Cottrell

**Affiliations:** Department of Biological Sciences, Seton Hall University, South Orange, NJ 07079, USA; jaymes.fuller@student.shu.edu (J.F.); katherine.lefferts@student.shu.edu (K.S.L.); pooja.shah2@student.shu.edu (P.S.)

**Keywords:** osteoblasts, osteoclasts, osteocytes, biochemical markers of bone turnover, three-dimensional bone organoid model

## Abstract

Here, we report on the development of a cost-effective, well-characterized three-dimensional (3D) model of bone homeostasis derived from commonly available stocks of immortalized murine cell lines and laboratory reagents. This 3D murine-cell-derived bone organoid model (3D-mcBOM) is adaptable to a range of contexts and can be used in conjunction with surrogates of osteoblast and osteoclast function to study cellular and molecular mechanisms that affect bone homeostasis in vitro or to augment in vivo models of physiology or disease. The 3D-mcBOM was established using a pre-osteoblast murine cell line, which was seeded into a hydrogel extracellular matrix (ECM) and differentiated into functional osteoblasts (OBs). The OBs mineralized the hydrogel ECM, leading to the deposition and consolidation of hydroxyapatite into bone-like organoids. Fourier-transform infrared (FTIR) spectroscopy confirmed that the mineralized matrix formed in the 3D-mcBOM was bone. The histological staining of 3D-mcBOM samples indicated a consistent rate of ECM mineralization. Type I collagen C-telopeptide (CTX1) analysis was used to evaluate the dynamics of OC differentiation and activity. Reliable 3D models of bone formation and homeostasis align with current ethical trends to reduce the use of animal models. This functional model of bone homeostasis provides a cost-effective model system using immortalized cell lines and easily procured supplemental compounds, which can be assessed by measuring surrogates of OB and OC function to study the effects of various stimuli in future experimental evaluations of bone homeostasis.

## 1. Introduction

Under homeostatic conditions, bone is continually modified and reshaped to maintain its critical mechanical and structural properties. This continual process is driven by bone depositing and bone-absorbing cells whose activity is tightly regulated. Osteoclasts are bone-resorbing cells that are derived from leukocytes of the monocyte/macrophage lineage. Osteoclastic progenitors are recruited to the bone from nearby bone marrow or as a result of local chemotactic stimuli [1,2]. Under the influence of soluble factors, such as receptor activator of nuclear factor kappa-B ligand (RANKL) and macrophage colony-stimulating factor (M-CSF), osteoclast progenitors differentiate into mature osteoclasts [3]. These osteoclasts secrete hydrogen ions (H+) that acidify the local environment and break down the mineral component of bone. Osteoclasts also secrete various peptidases and proteinases, which degrade the proteinaceous components of the bone matrix [1,2]. Together, these actions lead to the breakdown and resorption of bone matrix at the site of remodeling.

Osteoblasts are matrix-depositing cells that are derived from mesenchymal stromal/stem cells (MSCs) that migrate to the site of bone remodeling bone through the local soft tissue or peripheral vasculature. These osteoprogenitors then differentiate into mature osteoblasts under the influence of various factors including bone morphogenic proteins (BMPs) and phosphate-containing compounds which can serve as substrates for the alkaline phosphatase, which is a critical enzyme expressed by developing and mature osteoblasts [1,2,4,5,6]. Functionally mature osteoblasts group together and form interwoven clusters of cells which can adhere together via tight junctions. Osteoblasts promote hydroxyapatite formation within the local extracellular matrix to produce bone. When osteoblasts become surrounded by the bone matrix, they differentiate into osteocytes. Osteocytes maintain important mechano-sensing capabilities and regulate bone structure and remodeling in a load/stress-dependent manner [1,2]. 

Murine and human 3D organoid models have increasingly been developed to overcome some of the physiological limitations of in vitro 2D models [7,8]. Three-dimensional (3D) organoid models use various biomaterials to recreate the extracellular matrix and provide a more physiologically relevant environment [9,10,11,12]. These organoids are infused with cells that differentiate and will engage in self/material-induced organization [13]. Fully replicating the intricacies of bone is challenging, as it is a dynamic mineralized connective tissue composed of both inorganic hydroxyapatite and organic components such as collagen, proteins, and fats. Several 3D bone tissue models have been developed to study bone maturation and disease, but some are limited to a simplistic design, and models can vary in cell origin (i.e., primary tissue or pluripotent stem cells) [13,14,15,16], extracellular matrix material (Engelbreth–Holm–Swam matrix, demineralized bone matrix, hydroxyapatite) [17,18,19,20], and organoid type (i.e., callus, woven, cartilaginous, trabecular) [21,22,23,24,25,26]. In spite of these challenges, many initial organoid models have been successful at contributing to our understanding of bone biology [1,10,11,13,23,24,26,27,28,29,30,31,32,33]. And several bone organoids have been developed to recreate a bone-related disease, eliminating species differences and realistically mimicking the human osteoimmunological environment in conditions like osteoporosis [34], bone cancer [29,35], osteoarthritis [32,36] and genetic disease models [30]. In spite of the challenges with model standardization and precise recreation of the diverse and complex vascularized bone structure, these organoids provide a tool to complete basic research and clinical applications [1,10,11,13,23,24,26,27,28,29,30,31,32,33]. 

While the translational potential of 3D bone organoid models is high, there remains a further opportunity to reiterate this model using more cost-effective and broadly available immortal murine cell lines and common reagents without the need for complex equipment. In light of this, we set out to develop a cell-line derived murine 3D model of bone homeostasis (3D-mcBOM) derived from renewable stocks of murine cell lines (MC3T3-E1 and RAW 264.7) coupled with easy-to-measure surrogates of osteoblast and osteoclast function. This in vitro 3D-mcBOM enables the study of cellular and molecular mechanisms that regulate or disturb bone homeostasis, the data from which can be connected to in vivo mechanistic and translational studies in standard murine models of bone homeostasis and bone disease.

## 2. Results

### 2.1. MC3T3-E1 and RAW 264.7 Cells Differentiate into Functional Osteoblasts and Osteoclasts Induced by Osteoblastogenic and Osteoclastogenic Media

**Figure 1:**
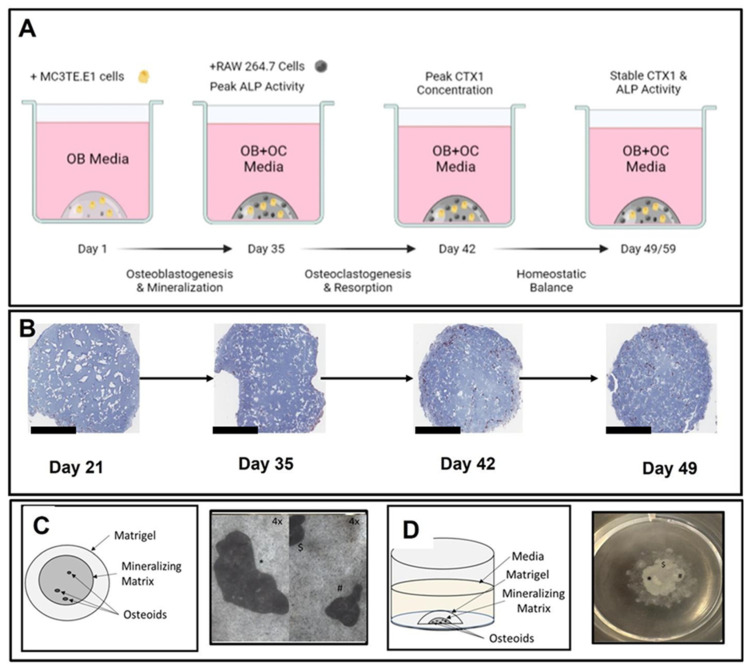
Animated, histological, and photographic schematic of 3D-mcBOM matrix generation. (**A**) Overview of in vitro model including major milestones and interventions. (**B**) Trichrome staining of 3D-mcBOM overtime (day 21 through day 49) magnification—2×, the bar—1 mM. ((**C**)-Left) Dorsal cartoon diagram of 3D bone culture; ((**C**)-Right) Photomicrographs of representative 3D bone culture following 21 days of osteoblastogenic differentiation with individual mineralized osteoid, 4× (*, $, #). ((**D**)-Left) Lateral cartoon diagram of 3D bone culture. ((**D**)-Right) Photomicrographs of representative 3D bone culture with mineralizing core contained within the Matrigel droplet inside a 22 mm diameter well, 1×.

The murine cell-line derived 3D-mcBOM described here leverages the ability to functionally differentiate cell lines into mature functional osteoblasts and osteoclasts resulting in mineral deposition and mineralization of the Matrigel matrix and resorption of the mineral and proteinaceous components of bone. Figure 1A,B depict the overall process for the generation of mineralized 3D bone fragments including the embedment of MC3TE-E1 cells in Matrigel and mineralization of the matrix. The mineralized fragment and osteoid forms in the center of the Matrigel droplet are depicted in Figure 1C,D.

**Figure 2:**
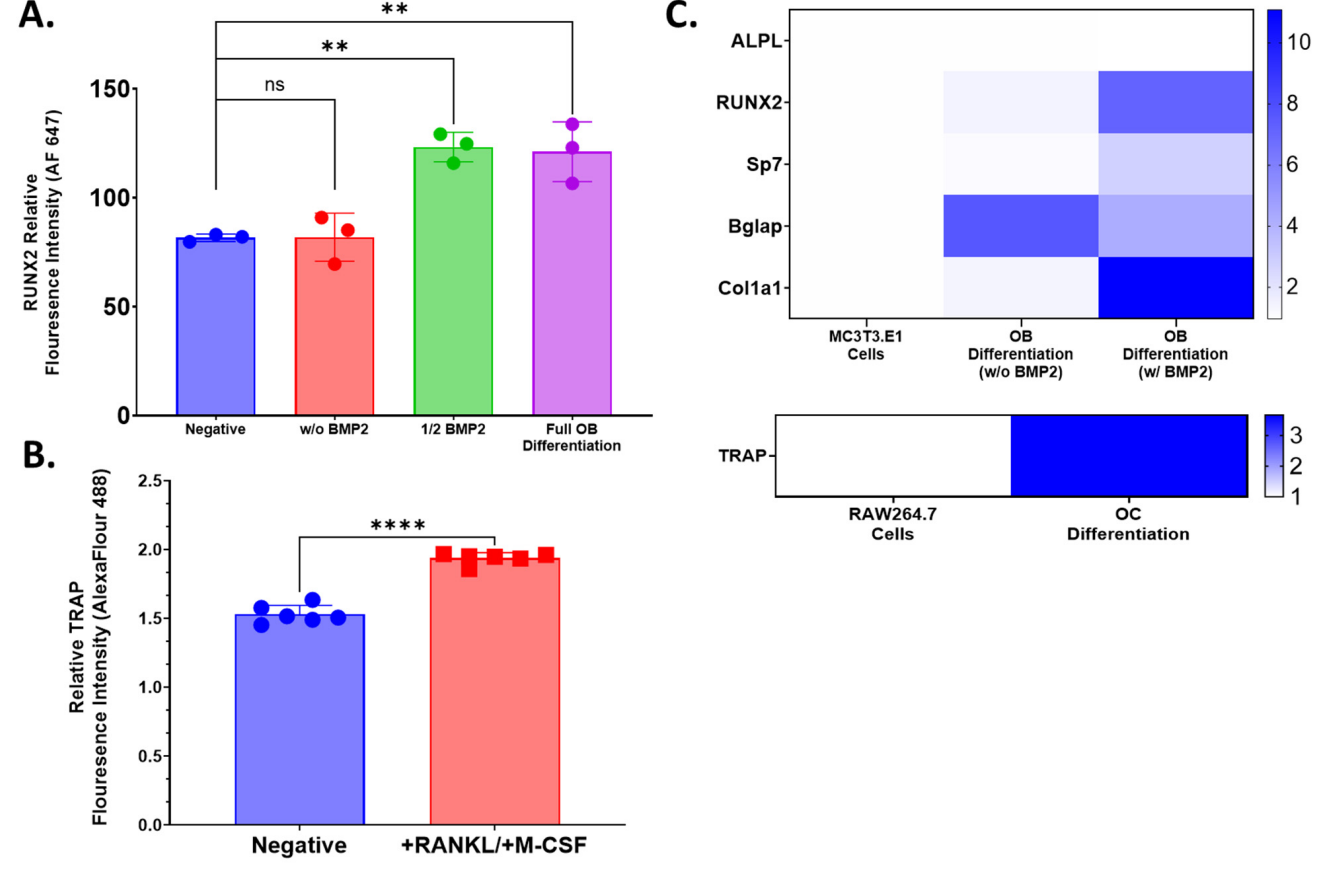
Relative expression screening of key osteoblast and osteoclast markers by in response to differentiation media. (**A**) RUNX2. (**B**) TRAP. Relative fluorescence intensity associated with protein expression evaluated from MC3T3-E1 and RAW 264.7 which were grown in 2D culture with exposure to media containing mixtures of osteoblast (BMP2) or osteoclast (RANKL/M-CSF) differentiating factors as compared to complete culture media (negative).. Fluorescence intensity for each condition was assessed by flow cytometry. Raw fluorescence data were normalized to a cell line specific unstained control for each group. Statistical significance was evaluated by one-way ANOVA (**A**,**B**) or unpaired T test (** = *p* < 0.01; **** = *p* < 0.0001). (**C**) Qualitative assessment of relative gene expression (2^−ΔΔCt^) of osteoblastogenic (*ALPL, Runx2, Sp7, Bglap, Col1a1*) and osteoclastogenic (*Trap*) genes of undifferentiated cell lines (pooled) versus cells differentiated (pooled) in two-dimensional culture for (MC3T3-E1: day 14; RAW 264.7: day 5). Where appropriate, data are summarized as the sample mean, and error bars represent the sample standard deviation.

Prior to beginning 3D cultures experiments, the osteoblastogenic and osteoclastogenic potency of the media was confirmed using two-dimensional cultures of RAW 264.7 and MC3T3-E1 cells. The MC3T3-E1 cells were cultured for 21 days, while RAW 264.7 cells were cultured for 5 days. Flow cytometry was used to evaluate the overall expression of runt-related transcription factor 2 (Runx2) and ALPL with respect to osteoblast differentiation and TRAP expression with respect to osteoclast differentiation. Exposure to osteoblastogenic media significantly increased levels of Runx2 in cell populations relative to the control (Figure 2A). Of note, the exclusion of BMP2 from the osteogenic media attenuated the increase in Runx2 levels, indicating that BMP2 is necessary for osteoblast differentiation within this in vitro system. Separately, cultures of RAW 264.7 in osteoclastogenic media resulted in a significant increase in levels of TRAP protein, indicating the phenotypic differentiation of RAW 264.7 cells to osteoclasts (Figure 2B). 

Phenotypic changes observed with exposure to differentiating conditions were mirrored in transcriptomic profiles of key osteoblastogenic and osteoclastogenic genes. The bone-related transcription factors, *Runx2* and *Sp7*, as well as the bone-related protein osteocalcin (*Bglap*), and collagen 1 alpha 1 (*Col1a1*) were all upregulated under osteoblastogenic conditions relative to the undifferentiated cell line control (Figure 2C). Consistent with the phenotypic analyses, levels of *ALPL* transcript were not impacted by osteoblastogenic conditioning. In parallel, osteoclastogenic conditioning was observed to have increased TRAP (*ACP5)* gene expression relative to undifferentiated RAW 264.7 cells (Figure 2C). Similar gene expression profiles were observed in 3D culture in both a time and cell-line dependent manner (Supplementary Figure S1).

### 2.2. Mineral and Protein Composition of 3D-mcBOMs Is Bone-like 

**Figure 3:**
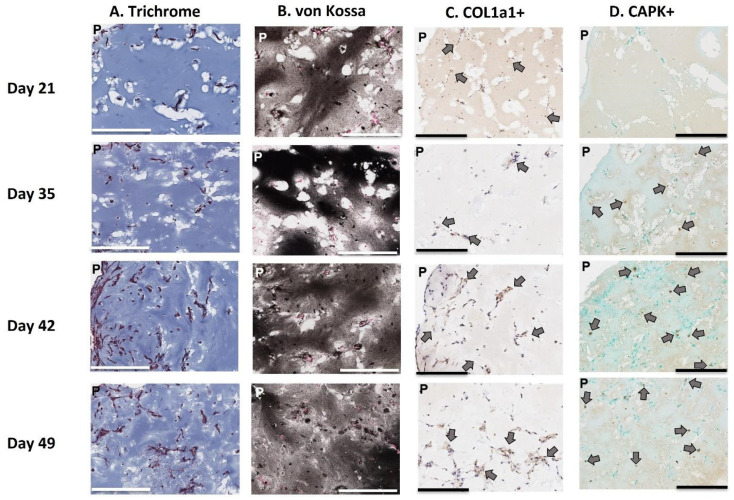
Histological staining of the 3D-mcBOM matrix cultures. Three-dimensional (3D) cultures were grown in 12-well plates in 128 μL drops (n = 6) and were harvested on days 21, 35, 42, 49 and stained/imaged as described above. Arrows indicate positive staining, P refers to the perimeter of the organoid, and the bar = 200 μM. Representative images are displayed in 10× for (**A**) Masson’s Trichrome staining, indicating mineralized collagen matrix (light to darker blue) with increasing proportion of embedded osteocyte-like cells over time. (**B**) depicts von Kossa calcium staining indicated by black area with cytoplasm and nuclei counterstained in red and dark brown, respectively. (**C**) Col1a1-positive stained cells in brown (**D**) Cathepsin K+-positive stained cells in brown.

Histological staining was conducted on 3D-mcBOM samples collected over the course of 28 days beginning after the 21st day of culture (days 21 to 49). Specifically, Masson’s trichrome staining revealed a consistent but increasing intensity of the mineralized extracellular matrix through the 28-day time course (Figure 1B and Figure 3A). A similar trend was observed with calcium-specific von Kossa staining indicating an increase in calcium deposition density over time (Figure 3B). Immunohistochemistry confirmed collagen1a1 (Col1a1) expression between days 21 and 49 and cathepsin K (CapK) between days 35 and 49 in the 3D-mcBOM organoids. Since the introduction of osteoclast precursor cells occurs after day 21, the absence of CapK on day 21 was expected and confirms that osteoblasts are expressing col1a1, secreting a mineralized matrix and depositing calcium throughout the 3D-mcBOM (Figure 3C,D).

**Figure 4:**
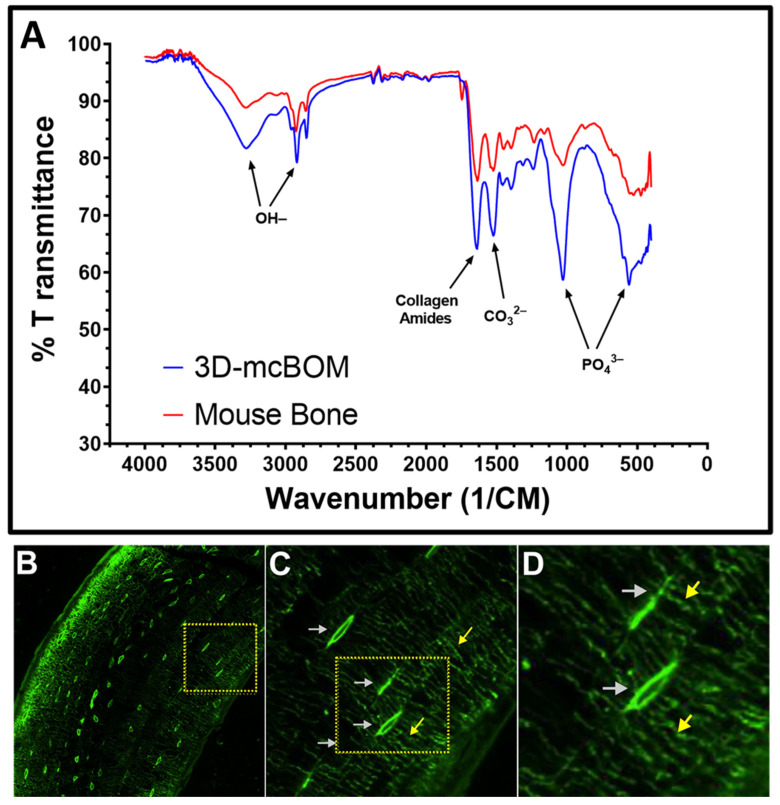
Structural analysis of 3D-mcBOM samples. (**A**) FTIR spectra of bone-like fragment (3D-mcBOM) produced from osteoblast-differentiated murine MC3T3.E1 cells (red) or femurs resected from adult mouse (blue) contain expected peaks at 500 and 1050 cm^−1^ associated with hydroxyapatite-derived phosphate groups and carbonate (1550 cm^−1^) as well as the collagen 1-related amide peak at approximately 1650 cm^−1^. (**B**) FITC staining of 3D-mcBOM Sample, 20×. (**C**) Enlarged area outlined in panel C shows osteocyte lacunae. (**D**) Enlarged area outlined in panel C shows canaliculi connecting osteocyte lacunae. Gray arrows identify Ot.Lc (Osteolytic Lacunae), and yellow arrows identify canaliculi.

Fourier-transform infrared (FTIR) spectroscopy confirmed that the material composition of 3D-mcBOM was similar to normal mouse bone. FTIR is a validated method to assess bone mineral content via the spectra transmittance profiles associated with hydroxyapatite and its key elemental compounds (phosphate and carbonate groups). The FTIR spectra of powdered 3D-mcBOM and mouse femur samples were analyzed (Figure 4A). The spectral transmittance profiles of 3D-mcBOM and mouse bone substantially overlap with significant regional similarity in spectra associated with PO_4_^3−^ and CO_3_^2−^ as well as primary collagen amide peaks associated bone matrix-associated collagen I. Hydroxyl peaks were also evident at higher wavenumbers for both samples. Additionally, the 3D-mcBOM bone microstructure was assessed using Ciani et al.’s method [37]. The layered bone structure, osteocyte lacunae, and canaliculi connecting the osteocyte lacunae can be readily observed in the FITC-stained 3D-mcBOM (Figure 4B, C, and D, respectively).

**Figure 5:**
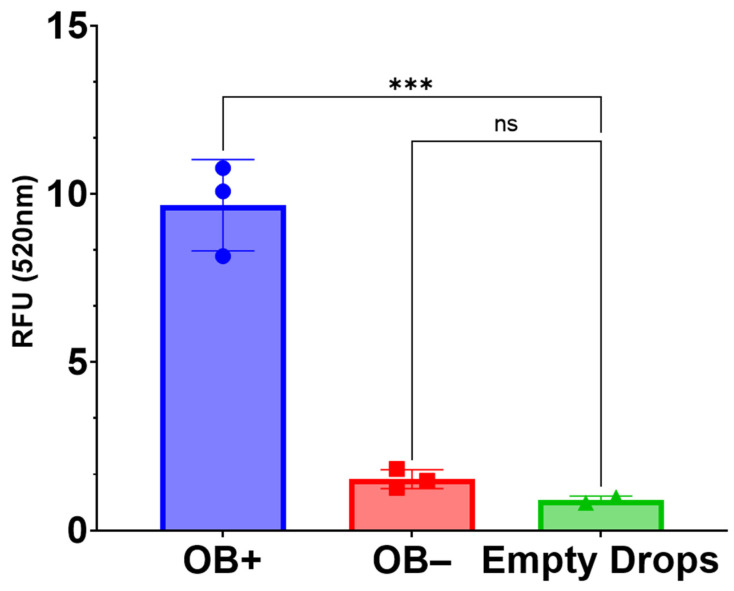
Hydroxyapatite content. OsteoImage assay was used to compare the hydroxyapatite content between 3D-mcBOM in osteoblastogenic differentiating conditions (OB+), MC3T3.E1 cells in Matrigel in complete media (OB−), or empty Matrigel drops complete media following 35 days of culture (*** = *p* < 0.001). Relative fluorescence increases proportionally to the levels of green-fluorescent, hydroxyapatite-specific OsteoImage dye retained in the sample after wash. Note that one of the three replicates for the control condition was lost during sample preparation. Data are summarized as the sample mean, and error bars represent the sample standard deviation.

To further evaluate the bone-matrix like nature of 3D-mcBOMs, the hydroxyapatite content was evaluated in 3D-mcBOM samples and controls. Bone mineral hydroxyapatite is the major mineral component of bone and is produced by osteoblasts to mineralize collagen fibers during bone formation and repair. As such, the production of hydroxyapatite is highly indicative of both functional osteoblast differentiation and the formation of a hard, mineralized bone matrix. Resultingly, the evaluation of hydroxyapatite content in 3D-mcBOMs by hydroxyapatite-specific fluorescent staining confirmed the production of bone minerals as compared to controls (Figure 5).

### 2.3. Kinetics of Bone Formation and Resorption Indicators Responds to Differentiation Conditions

**Figure 6:**
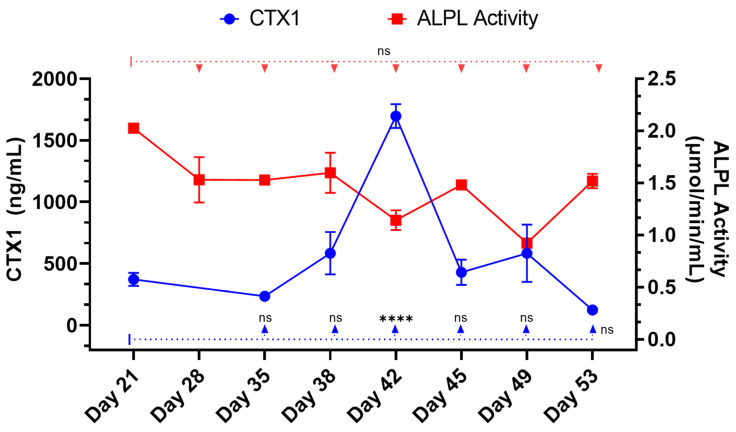
Kinetics of ALPL activity and CTX1 concentration during 3D-mcBOM matrix development. Kinetic analysis of alkaline phosphatase activity (μmol/mL/min) and CTX1 concentration (ng/mL) from clarified cell culture supernatant from 3D bone cultures (n = 5 for ALPL; n = 4 for CTX1). Two-way ANOVA with Dunnett’s multiple comparison test was used to compare levels of ALPL activity and CTX1 concentration over a time course of model maturation relative to Day 21 (**** = *p* < 0.0001). Data are summarized as the sample mean, and error bars represent the sample standard deviation.

Alkaline phosphatase (ALPL) in clarified supernatant from 3D bone cultures was measured as an indicator of osteoblast activity (Figure 6). Alkaline phosphatase levels were generally consistent between 21 and 28 days (1600 and 1250 ng/mL, respectively) with no statistical difference between these two days. Following the addition of RAW 264.7 cells on day 35, changes in ALPL and type I collagen cross-linked telopeptide (CTX1) levels in the 3D-mcBOM culture media were most notable on days 38 and 42. During this period, the RAW 264.7 cells appear to have matured into differentiated osteoclast with CTX1 release on day 42 (151% change over day 35, *p* value: 0.0001). Following this period, the levels of ALPL and CTX1 stabilize as osteoblast mineralization activity and matrix resorption re-establish a balance between mineralization and resorption (Figure 6, days 45–53). 

**Figure 7:**
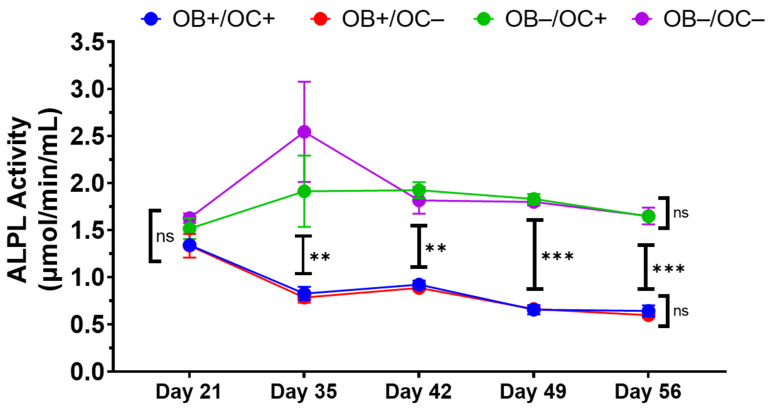
Alkaline phosphatase activity inversely correlates with mineralization. Kinetic quantitative analysis of alkaline phosphatase activity in supernatant from 3D-mcBOMs. OB+: with OB differentiation media supplements added, OB−: without OB differentiation media supplements, OC+: with exogenous OC differentiation supplements added, OC−: without OC differentiation media supplements (N = 3 per group per time point). Statistical significance was determined within and across OB and OC groups at each time point via two-way ANOVA with Tukey’s multiple comparison post hoc testing (** = *p* < 0.01; *** = *p* < 0.001). Data are summarized as the sample mean, and error bars represent the sample standard deviation.

To further characterize the kinetics of ALPL activity as a surrogate of osteoblastogenesis and osteoblast activity, MC3T3-E1 cells embedded in Matrigel were exposed to media containing or lacking OB and/or OC exogenous differentiating supplements. Culture supernatants were evaluated for ALPL activity over a 34-day time course from days 21 to 56 (Figure 7). ALPL activity levels for all conditions were comparable at day 21 and appeared to increase at later times without OB differentiation media supplements (OB−). Conversely, ALPL activity levels significantly decreased from day 21 in MC3T3-E1-Matrigel cultures exposed to OB differentiating media supplements (OB+). Despite the differential kinetics of ALPL activity across OB differentiating and non-differentiating conditions, the kinetic profile within each condition group (+ or −) was not found to be statistically different across the entire time course.

**Figure 8:**
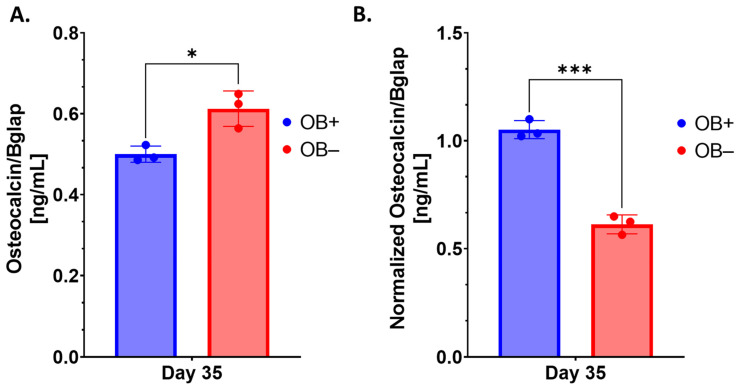
Assessment of relative osteocalcin concentrations. Original (**A**) and viability/protein concentration-normalized (**B**) levels of osteocalcin concentration in supernatant from 3D-mcBOMs. OB+ (with OB differentiation media supplements added or OB− (without OB differentiation media supplements) (N = 3 per group). Statistical significance was determined within and across OB and OC groups at each time point via two-way ANOVA with Tukey’s multiple comparison post hoc testing (* = *p* < 0.05; *** = *p* < 0.001). Normalization of osteocalcin concentrations was performed by dividing the pooled ratio for differences in viability and protein concentration (Supplementary Figure S2) between OB+ and OB− conditions. Data are summarized as the sample mean, and error bars represent the sample standard deviation.

To better understand the kinetics of osteoblastogenic indicators, cell viability and total protein concentration were evaluated in OB-differentiating (OB+) or non-differentiation (OB−) conditions at Day 35. Assessment of cell viability indicated a statistically significant decrease in cell activity in the OB+ conditions relative to the OB− conditions (Supplementary Figure S2A). However, the total protein concentration was found to be generally similar across the two conditions at day 35 (Supplementary Figure S2B), suggesting an overall increased rate of protein production in the OB+ group (more protein mass per active cell resulting in similar protein levels). In this context, osteocalcin levels were measured in the supernatant from each condition. As expected, osteocalcin concentrations were found to be lower in the OB+ group relative to the OB− group (Figure 8A); however, when corrected for differences in cell viability and protein concentration, normalized concentrations of osteocalcin were found to increase in the OB+ group relative to the OB+ group (Figure 8B), which is consistent with the qualitative gene expression analysis described earlier.

### 2.4. Mineralization Is Required and Independently Sufficient to Induce Osteoclastogenesis and Matrix Resorption

**Figure 9:**
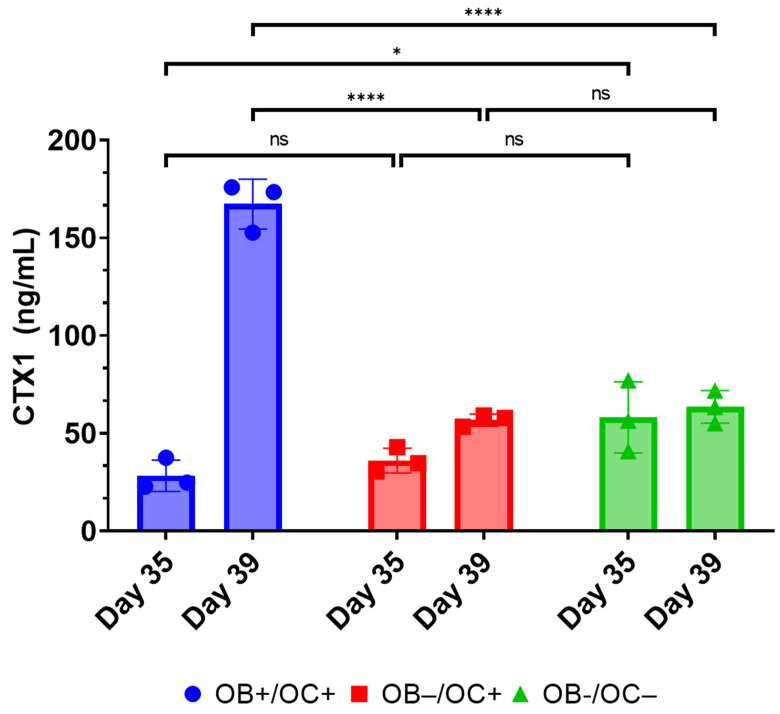
Comparison of CTX1 levels in context of matrix mineralization. Quantitative analysis comparing levels of CTX1 in media supernatant collected from cultures exposed to osteoblast differentiation or non-differentiating condition (n = 3). OB+: with OB differentiation media supplements added, OB−: without OB differentiation media supplements, OC+: with exogenous OC differentiation supplements added, OC−: without OC differentiation media supplements. Statistical significance was determined by two-way ANOVA with Tukey’s post hoc testing (* = *p* < 0.05, **** = *p* < 0.0001). Data are summarized as the sample mean, and error bars represent the sample standard deviation.

Next, we sought to better understand the dynamics and kinetics of osteoblast and osteoclast differentiation and activity. CTX1 concentrations were determined, by ELISA, from clarified supernatants harvested from cultures exposed to both OB with OC differentiating conditions, OC differentiating conditions, or control media at days 35 and 39 (Figure 9). A dramatic increase in CTX1 levels was observed between days 35 (the point of RAW 264.7 cell addition) and day 39 but only within the pro-osteoblastogenic condition (OB+/OC+). No substantial increase in CTX1 was observed in the OB− conditions even in the presence of OC-differentiating factors (OC+). These results indicate that OB+ conditions are necessary for osteoblast-dependent synthesis of type I collagen as measured by osteoclast-dependent release of the CTX1 peptide.

**Figure 10:**
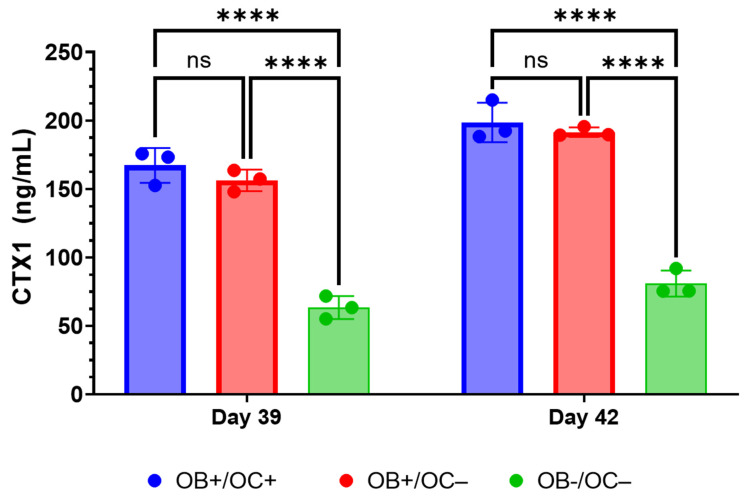
Three-dimensional (3D) bone model induces osteoclastogenesis without addition of osteoclastogenic supplements. Quantitative analysis comparing levels CTX1 concentrations (ng/mL) following the addition of RAW 264.7 cells to 3D bone cultures with (OB+/OC+) and without (OB+/OC−) osteoclastogenic supplements relative to negative control (OB−/OC−) in the presence of mineralized 3D-mcBOM (n = 3). Statistical significance was defined based on a two-way ANOVA with Tukey’s post hoc multiple comparisons (**** = *p* <0.0001). Data are summarized as the sample mean, and error bars represent the sample standard deviation.

To determine whether the osteoblasts within the 3D-mcBOM cultures were sufficient to promote osteoclast differentiation, CTX1 levels were measured in media from 3D-mcBOM cultures exposed to either OC differentiating or non-OC differentiating media (Figure 10). Despite the lack of supplemented RANK-L and M-CSF, CTX1 levels in the non-OC differentiating condition increased following RAW 264.7 additions to levels comparable to those in the OC differentiating conditions, while CTX1 levels overall were significantly higher in these two conditions relative to the negative control (OB−/OC−). 

**Figure 11:**
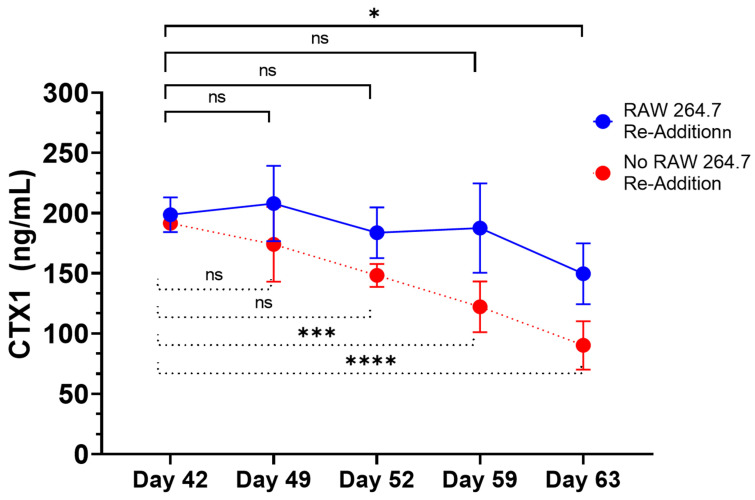
Re-addition of RAW 267.4 cells prolongs homeostatic balance of bone resorption in 3D-mcBOM. Quantitative analysis comparing levels of CTX1 levels in culture supernatant following re-addition of RAW 264.7 cells (day 42). Comparisons were restricted to these conditions while negative control was plotted as a qualitative reference. Statistical significance was determined by two-way ANOVA with Tukey’s post hoc testing to day 42 as reference control for each condition with N = 3 replicates per group per time point (* = *p* < 0.05; *** = *p* < 0.001, **** *p* < 0.0001). Data are summarized as the sample mean and error bars represent the sample standard deviation.

We also assessed the functional lifespan of osteoclasts based on the stability of the CTX1 signal over time. In this context, we also re-introduced RAW264.7 cells at the time point previously associated with peak CTX1 levels and compared this condition to one in which RAW 264.7 cells were only introduced at day 35 (Figure 11). The re-addition of RAW 264.7 cells appeared to prolong the stability of peak CTX1 concentrations to 59 days from the day 42 peak and had statistically significant differences in CTX1 levels emerge at day 52 when RAW 264.7 cells were only added at day 35. Of note, CTX1 levels were remarkably stable between day 42 and day 52 with a range of 207.6 to 183.6 ng/mL and difference of 24.3 ng/mL, which is comparable to the mean standard deviation for replicate cultures throughout the dataset (25.8 ng/mL). Resultantly, the kinetics of CTX1 with and without cell re-introduction suggests a stable functional lifespan of approximately 7 days in regard to osteoclast activity. 

## 3. Discussion

The use of animal models in biomedical research facilitates the evaluation of biological processes based on in vivo biology [18,23,27,38]. Translating in vitro observations into complete physiological systems using animal models is a long-standing practice. However, the complexity, cost, and ethical considerations of conducting studies in relevant animal models can be prohibitive [38]. Developing relevant animal models is time and resource intensive, as is the maintenance and husbandry of animal colonies. In vivo models, particularly large organism models most associated with research relevant to humans, have historically low experimental throughput, further increasing cost and time. Ethical considerations to limit the number of animals used in biomedical research require the development of appropriate in vitro model systems. In this context, the development of biologically representative in vitro models is a resourceful and ethically effective approach to studying key physiological systems. While current technology limits the full representativeness of cell-based in vitro models to full organisms, these models may be used as a bridge and complement to extend, validate, or prioritize experimentation in animals [1,2,10,13,14,16,22,23,29,39].

Previous studies spanning many different physiological systems have demonstrated the differential behavior of in vitro and ex vivo models developed in 2D or 3D geometry. The evaluation of model systems in lung, intestinal, or neuronal settings has highlighted the significantly differentiated gene expression profiles of cells grown in 2D versus 3D [1,2,9,10,11,12,13,14,16,20,22,23,24,26,28,29,32,33,35,39,40,41]. Furthermore, experimental outcomes highlight differences in behavior and responsiveness to stimulate in 2D models relative to 3D models. Specifically in the context of bone, Tortelli et al. demonstrated the differential expression of key genes regulating the differentiation and maturation of osteoblasts between 2D and 3D models. This differential profile resulted in the development of a more “physiological bone”-like structure formation in 3D models [33]. Barron et al. further demonstrated relevant physiological differences by highlighting changes in osteoblast gene expression under both static and mechano-dynamic conditions [42]. 

In light of these considerations, our objective was to develop a 3D model that sufficiently represents the differentiation and behavior of key bone-related cells and the mineral and protein content of the bone matrix. Importantly, we established the model by leveraging readily available and relatively inexpensive materials (ECM-supplemented hydrogel matrices, chemical supplements, recombinantly expressed proteins). Additionally, we endeavored to use established murine cell lines that can be propagated perpetually versus isolated murine primary cells which have limited lifespans and must be routinely collected from euthanized animals. 

The 3D-mcBOM, as established here, consists of mouse MC3T3.-E1 embedded in a hydrogel matrix (Matrigel) and induced to differentiate into osteoblasts and elaborate a mineralized matrix before the addition and differentiation of mouse RAW 264.7 cells into osteoclasts [13,41,43,44,45]. The hydrogel matrix is derived from basement membrane extracts of Engelbreth–Holm–Swarn mouse sarcoma and contains major cellular matrix (ECM) components including laminins, collagen IV, heparan sulfate proteoglycans as well as growth factors (TGFβ, epidermal growth factor (EGF), insulin-like growth factor (IGF) and fibroblast growth factor (FGF) [46,47,48,49]. This combination of ECM and growth-promoting proteins is able to sustain cell persistence/expansion; however, it is insufficient to induce the functional differentiation of MC3T3.E1 or RAW 264.7 cells in the absence of OC and OB-differentiating factors. Additionally, Matrigel remains liquid when maintained between 2 and 8 °C, allowing for the easy manipulation and integration of other material such as cells, cytokines, growth factors, etc. The liquid matrix then rapidly polymerizes into a solid when incubated at 37 °C and remains stable for up to 12 weeks based on our experience. The properties create the ideal three-dimensional matrix to manipulate and characterize the activity and behavior of cells under specific conditions related to osteoblastogenesis and osteoclastogenesis. Specifically, 3D cultures and the differentiation of bone-related cells has been shown to enhance the expression of bone-related proteins [48].

Like many 3D bone organoids, this 3D-mcBOM does not fully mimic in vivo bone and has limitations including the lack of vascularization and the exact physical structure of bone [1,13]. However, this 3D-mcBOM culture does produce a bone-like mineralized matrix structure, synthesize type I collagen, and produce hydroxyapatite. Interestingly, the profile of ALPL observed in osteoblastogenic conditions was unexpected. Izumiya et al. have evaluated the characteristics of MC3T3.E1 cells in response to various osteoblast-differentiating conditions and media formulation and observed that the presence of vitamin C (L-ascorbic acid) can meaningfully influence ALPL gene expression and ALPL activity. Specifically, in the presence of ascorbic acid, ALPL activity from treated MC3T3.E1 cells was found to be generally comparable between control and osteoblastogenic media conditions while still promoting calcification in response to osteoclastogenic conditions [45]. Other indicators of osteoblastogenesis, such as osteocalcin, may also be considered as key analytes, assuming that the variation in cell viability and total protein content are taken into account. Regulated apoptotic cell death in osteoblasts/osteocytes has previously been characterized and is a process that reflects natural bone formation and metabolism [44]. As such, the observation of decreased cell viability on 3D-mcBOM cultures relative to non-mineralized controls is likely indicative of this expected physiological process; however, the presence of active viable cells in 3D-mcBOMs does potentially suggest a renewable reservoir of osteoblast-like cells which could sustain long-term homeostasis as described above.

Furthermore, the RAW264.7 osteoclasts within the 3D-mcBOM produce TRAP and appear to secrete proteases that can release CTX1 from type I collagen [43]. Once established, the 3D-mcBOM can support osteoclast differentiation without exogenous osteoclastogenic factors and osteoblast differentiation without exogenous osteoblastogenic factors. Previously, the culture of pre-osteoblast-like cells lines on mineralized matrix or bone has been demonstrated to induce osteoblastogenesis [41,42,44,45]. Additionally, functional osteoblasts are a physiologically relevant source of RANKL, which may prompt the differentiation and function of osteoclasts as part of normal bone homeostasis [28]. While RANKL and M-CSF are broadly described in the literature with respect to their ability to promote the in vitro differentiation of osteoclasts, the present data suggest that osteoblasts within the 3D-mcBOM can promote osteoclast differentiation, which may facilitate further model simplification and cost-reduction (elimination of recombinant RANKL and M-CSF). The ability of mature 3D-mcBOM to support osteoblastogenesis and osteoclastogenesis without additional factors may enable experiments to determine the effects of physiological and pathological stressors, such as hyperinsulinemia, hyperglycemia, LPS, or hypoxia, on bone homeostasis.

In describing the 3D-mcBOM, we acknowledge the limitations of the model as presented where the use of immortalized tumor-derived monoclonal cell lines may not fully recapitulate the complex interactions, autonomous localization, and physiological behavior of heterogenous mixtures of MSC-derived bone-related cells and stroma. To address this potential gap, additional experiments are planned to introduce other cell types also found in bone-related tissues (cartilage, vasculature, stroma) and study its response to clinically-relevant stimuli as well. As depicted above (Figure 1), the model produces an osteoid that is representative of the bone matrix in terms of cellular and molecular mechanisms sufficient to evaluate homeostasis (deposition and resorption). Additional optimization is being explored, including the model scale-up necessary to accommodate structural testing consistent with methods used to characterize bone derived from animals or human subjects. With all experimental models, variability derived from sources like changes in key reagents lot numbers, or environmental conditions associated with cell culture, can impede the ability of research to interpret results.

Other in vitro/ex vivo model systems of bone have been reported and are being evaluated as experimental systems or for regenerative medicine applications; however, many of these systems require complex bioreactors and other instrumentations that may limit the accessibility and sustainability of the model for broad use. In this context, leveraging immortalized cell lines and simple, readily available, well-controlled reagents creates an opportunity to introduce additional control over potential sources of variability within the proposed 3D-mcBOM. In this regard, cell-based platforms offer broader accessibility, reduced cost, and accelerated experimental turnaround times while reducing the use of animal models [1,9,13,38]. Thus, in this work, we have described the development and behavior of a 3D model of bone formation and homeostasis derived from immortalized cell lines and readily available reagents. The model system includes measurable surrogates of osteoblast (ALPL) and osteoclast (CTX1) activity [50,51]. CTX1 is the c-terminal peptide of type I collagen released from mature collagen matrix by osteoclast activity [50]. These chemical and mineral signatures of bone matrix serve as test variables in experimental systems seeking to evaluate bone homeostasis in response to various stimuli. The ability of mature 3D-mcBOM to support osteoblastogenesis and osteoclastogenesis without additional factors may enable experiments to determine the effects of physiological and pathological stressors, such as hyperinsulinemia, hyperglycemia, LPS, or hypoxia, on bone homeostasis. In the future, our lab would like to complete a 3D-mcBOM cross-validation to an acceptable animal model. 

While many organoid models are constructed using primary cell lines, our utilization of immortalized cell lines diverges from contemporary literature norms. Despite potential critiques of immortalized cells lacking some primary cell characteristics, our study convincingly demonstrates the functional replication of key bone traits. Importantly, our research broadens the scope of organoid model possibilities beyond primary cell types. Although organoids are not exact replicas of their respective organs, they carry functional and structural resemblances with a degree of fidelity. In this regard, our 3D-mcBOM fulfills the criteria of an organoid, representing bone homeostasis while acknowledging differences compared to in vivo bone and ex vivo cultures. Derived from immortalized pre-osteoblast-like MSC progenitors and monocyte-like cells, our model does not fully replicate the heterogeneity of physiological bone progenitors; however, various studies also document limitations associated with the use of primary or stem cell populations in organoids [1,2,13,52]. In our 3D-mcBOM immortalized progenitors, cells proliferate and differentiate into osteoblasts and osteoclasts, respectively, which effectively produces a mineralized matrix similar in composition to bone. All in vitro cultures lack the complete network of regulatory elements necessary to achieve and maintain bone homeostasis, such as signals from local stroma, endocrine hormones, the nervous system, and mechanical forces found in in vivo bone.

While it is true that our model does not replicate or reflect all of these physiological characteristics, we have demonstrated that the model is able to produce a mineralized matrix similar in chemical and protein composition to bone and corresponding to the detection of common biomarkers of bone metabolism and turnover. Via both confocal microscopy and histological staining, our model demonstrated that osteoblast cells are distributed throughout the entire 3D matrix, and as a result, the entire matrix is mineralized with bone-like chemical and protein composition. Second, we provide evidence that osteoclasts can migrate into and locally digest the matrix. Third, through FITC staining, we revealed osteocyte lacunae within the 3D-mcBOM, which offers evidence of the differentiation of osteoblasts into osteocytes. Finally, the FITC imaging indicates that these immortalized cells self-assembled and developed a structure with similar morphology to normal bone. Given this, future studies using our model may be able to provide insight into osteocyte signaling to osteoclasts and osteoblasts.

The use of immortalized cell lines offers experimental consistency and a longer lifespan, facilitating the bridging of various in vitro and in vivo studies. Additionally, the model’s adaptability to fluorescent tagging and visualization techniques opens avenues for more detailed mechanistic investigations into bone-related processes within the 3D structure. Further development of the 3D-mcBOM platform may be further explored by mixing immortalized cells with primary cells to reduce system variability, such as for example, using immortalized MC3T3-E1 cells with primary bone marrow monocytes, or by including additional immortalized or primary cell types such as endothelial cells or chondrocytes. The use of more structurally organized basal matrices to support a complex three-dimensional structure may also be considered in parallel with both exposure to mechanical forces as a development stimuli and the characterization of biomechanical and physical properties. In light of these future research directions, the 3D-mcBOM described above is a suitable representation of bone metabolism in terms of matrix deposition and resorption, which is consistent with the original hypothesis and objective of the research work.

## 4. Materials and Method

### 4.1. Culturing MC3T3-E1 Pre-Osteoblasts and RAW264.7 Monocytes

The murine pre-osteoblast cell line, MC3T3-E1 Subclone 4, and murine monocyte cell line, RAW 264.7, were acquired from the American Type Culture Collection (ATCC; Manassas, Virginia Catalogue# MC3T3.31: CRL-2593 and RAW264.7: TIB-71) as a cryogenically preserved cell suspension. Thawed cells were recovered and cultured in Alpha Minimal Essential Medium (αMEM) (Corning Inc. Catalog#10-022-CV, Union City, CA, USA or Thermo Fisher Inc. Gibco Catalog #41061029, Waltman, MA, USA) supplemented with 10% heat-inactivated fetal bovine serum (FBS) and 1% 100X penicillin/streptomycin (Corning Inc. Catalogue# 30-002-CI, Union City, CA, USA) at 37 °C (αMEM complete) with 5% CO_2_ in 100% relative humidity in standard tissue culture-treated vessels. Cultured MC3T3-E1 cells were passaged after achieving 70–85% confluence (3–4 days) using 0.05% Trypsin (Thermo Fisher Gibco Catalogue# 25300054, Waltman, MA, USA) or 1x TrypLE Express (Thermo Fisher Gibco Catalogue# 12605010, Waltman, MA, USA). Cultured RAW264.7 cells were passaged by scraping every 3 to 4 days or when adherent colonies of cells were apparent. 

### 4.2. Generation of 3D Bone Organoid Model

Osteoblastogenic (OB) media was composed of complete Alpha MEM supplemented with 100 ng/mL BMP-2 (R&D Systems Cat. Number 355-BM, Minneapolis, MN, USA), 15 mM beta-glycerophosphate (Sigma-Aldrich Cat. Number G9422, St. Louis, MO, USA), 100 μM L-ascorbic acid, and 100 nM dexamethasone (Sigma-Aldrich Cat. Number D2915, St Louis, MO, USA). Osteoclastogenic (OC) media was composed of complete Alpha MEM supplemented with 33.3 ng/mL RANK-L (R&D Systems Cat. Number 462-TEC-010, Minneapolis, MN) and 16.7 ng/mL M-CSF (R&D Systems Cat. Number 416-ML-050/CF), Minneapolis, MN. OB + OC media was also prepared and represents supplementation with all the osteoblastogenic and osteoclastogenic agents at the concentrations described above. Negative or vehicle control media was complete Alpha MEM only.

To generate matrix-stabilized 3D cultures, cultured MC3TE-E1 cells were trypsinized as described above, pelleted by centrifugation (500× *g* for 5 min at 2–8 °C) and resuspended in cold Growth Factor Reduced, Phenol Red Free Matrigel Matrix (Corning, Inc., Corning, NY, USA; cat# 35623) at a ratio of ~60,000 MC3TE-E1 cells to 16 μL of cold Matrigel. Matrigel, cells, Matrigel-suspended cells, pipette tips and serological pipettes were kept on ice during plating procedures to ensure that the Matrigel matrix did not prematurely polymerize. Then, 16 μL or 128 μL drops of cold Matrigel-suspended cells were carefully pipetted into the center of a well of a 96- or 12-well tissue culture treated plate, respectively. Of note, differences in culture scale may impact the relative concentration of key analytes of interest. Culture plates were subsequently incubated at 37 °C with 5% CO_2_ in an incubator with 100% relative humidity for 15–30 min to polymerize the Matrigel matrix and embed the MC3T3-E1 cells. Following matrix polymerization, OB media was added to all wells. Negative controls were treated with complete media. During the mineralization phase (day 0 to day 35), ½ OB media volumes were exchanged with fresh OB media every 3 to 4 days. 

At day 35, all media was removed from relevant wells, and RAW 264.7 cells suspended in OB + OC media were added to cultures at a target cell number of 60,000 or 480,000 cells per well of a 96-well or 12-well plate, respectively, and equal to the original number of MC3TE-E1 cells embedded in the Matrigel matrix. Cultures were maintained as indicated and unless otherwise specified for a maximum of 56 days unless otherwise indicated in experimental results. A summary detailing the principal experimental outcomes achieved at key milestones in the generation of 3D-mcBOM, along with corresponding figures, is provided in Supplementary Table S1. Experiments used 3 biological replicates where each biological replicate represents one cultured 3D-mcBOM culture.

### 4.3. Flow Cytometry

Flow cytometry was used to evaluate the expression of osteoblast lineage marker, Runx2, and osteoclast lineage marker, TRAP, following 14 days of differentiation in respective media to confirm the potency of those media formulations. Differentiated or control cells were isolated by trypsin digestion and repeated pipetting into single-cell suspensions. Cells were centrifuged (5 min, 500× *g* at room temperature) in PBS and resuspended in 1x True-Nuclear Fixation buffer (Biolegend, Cat. Number 424401, San Diego, CA, USA) for 60 min at room temperature. Then, 1 mL of 1x Perm Buffer (Biolegend, Cat. Number 424401, San Diego, CA, USA) was added to all tubes, which were washed as described above. Cell pellets were resuspended in 100 μL 1x Perm Buffer. Vendor-recommended volumes of stain antibodies were added to relevant tubes: 2.5 μL anti-TRAP Alexa Fluor 488 (Abcam Cat. No. Ab216934, Boston, MA, USA) for osteoclasts or RAW 264.7 control cells or 2.5 μL anti-Runx2 Alexa Fluor 647 (Abcam Cat. No. Ab215955, Boston, MA, USA) to osteoblasts or MC3T3.E1 cells. Unstained cells were resuspended in 1x Perm Buffer only. All tubes were incubated for 30 min at room temperature in the dark and subsequently washed with 2 mL of 1x Perm buffer followed by a second wash with 2% FBS in PBS (FACS Buffer). Cell pellets were resuspended in 500 μL with data acquisition on MASC Quant 7 (Milteny Biotec, Gaithersburg, MD, USA) and flow cytometry analysis using FlowJo (Treestar, Ashland, OR, USA). Fluorescence intensity values for cell populations and condition groups were normalized to unstained control. Cell culture and flow cytometry acquisition was performed with either 3 or 6 biological replicates. Statistical analysis was performed using GraphPad Prism 9.0. One-way ANOVA or an unpaired T-test was used to establish the statistical significance of comparisons. 

### 4.4. Qualitative Relative qPCR

RNA was extracted from pooled cultured primary cells, pooled 3D-mcBOM, or control samples using the QIAGEN RNeasy RNA (QIAGEN, Valencia, CA, USA; cat#74134) via the manufacturer-provided protocol. The quantity and purity of the extracted RNA samples were measured with a BioDrop Duo spectrophotometer (BioDrop Ltd.; Cambridge, UK). Extracted RNA was converted to complementary DNA (cDNA) with a Thermo Fisher Scientific High-Capacity cDNA Reverse Transcription Kit (Thermo Fisher Scientific; Waltman, MA, USA; cat#4368814) using the manufacturer’s thermocycler settings recommended for reverse transcription (RT). The resulting cDNA concentration and purity were measured with a BioDrop Duo spectrophotometer. TaqMan Fast Advanced Master Mix (Applied Biosystems, FosterCity, CA, USA; cat# 4444557) was mixed with nuclease-free water to 1x concentration. The appropriate volume of individual 20× TaqMan assays was added to achieve a 1× final concentration. The appropriate volume of validated individual 20× Thermo Fisher Scientific TaqMan assays was added to achieve a 1x final concentration (ALPL-Mm00475834_m1; RUNX2- Mm00501584_m1; SP7-Mm04933803_m1; BGLAP-Mm03413826_mH; COL1A1-Mm00801666_g1; TRAP-Mm00475698_m1; GAPDH-Mm99999915_g1). The complete master mix was then loaded into individual wells of a MicroAmp 384 well optical plate (Applied Biosystems, FosterCity, CA, USA; cat# 4309849). Then, 2 μL of cDNA per sample was added to relevant wells, and the plates were sealed and run on a 7900 HT Fast 384 Block polymerase chain reaction (PCR) system (Thermo Fisher Scientific, Waltman, MA, USA). Relative gene expression was calculated based on the 2^−ΔΔCt^ method with GAPDH as the endogenous control/calibrator.

### 4.5. Murine CTX1 ELISA

Type 1 Collagen Cross-Linked C Telopeptide (CTX1) ELISA assays (Novus Biologicals, Catalogue #NBP2-69074, Centennial, CO, USA or Biomatik Catalogue # EKF57814, Wilmington, DE, USA) were performed per the vendor-supplied protocol to measure the cell culture supernatant levels of CTX1, which is an indicator of bone resorption in the 3D-mcBOM. Briefly, cell cultures supernatants were collected, clarified by centrifugation (1000× *g* for 5 min), frozen, and stored at −80 °C until needed. Prior to the assay, frozen supernatants were thawed at room temperature and were diluted 400–500× in sample diluent based on method optimization. Samples and reconstituted standards were added to capture antibody pre-coated plates and incubated at 37 °C for 90 min. Following incubation, plates were washed 3–4 times with wash buffer, after which reconstituted biotin-labeled antibody was added with a subsequent 60-min incubation at 37 °C. Excess biotinylated antibody was decanted, and plates were washed as above with the subsequent addition of streptavidin-conjugated horseradish peroxidase followed by a 30-min incubation. Following incubation, the plate was washed as above, which was followed by the addition of TMB substrate. The colorimetric reaction proceeded at 37 °C for 30 min before the addition of stop solution. Optical density (OD) was read at 450 nm without wavelength path correction using a Varioskan LUX plate reader (Thermo Fisher Scientific Inc., Waltman, MA, USA). Curve fitting was conducted using SkanIt RE 6.1 (Thermo Fisher Scientific Inc., Waltman, MA, USA) with a 4-parameter logistic fit model of background-corrected raw OD values. Concentrations (ng/mL) of CTX1 were subsequently corrected for dilution with subsequent graphical and statistical analysis conducted in GraphPad Prism 9.0. 

### 4.6. Alkaline Phosphatase Activity

An alkaline phosphatase activity assay (Abcam, Catalogue# ab83369, Boston, MA, USA) was performed per the vendor-supplied protocol to measure alkaline phosphatase activity in cell culture supernatants of 3D-mcBOM samples as a surrogate of functional osteoblast activity. Briefly, collected cell cultures supernatants were clarified by centrifugation (1000× *g* for 5 min) and subsequently frozen and stored at −80 °C until needed. Frozen supernatants were thawed at room temperature, and undiluted aliquots of the supernatant were added to clear-bottom 96-well assay plates. Then, 5 mM pNPP substrate was added to the supernatant aliquots and standard wells. An alkaline phosphatase enzyme was then added to the standard wells over a range of concentrations (10, 20, 30, 40, 50 nmol/well in sample buffer) to create the standard curve. Assay plates were incubated at 25 °C for 60 min after which reaction was stopped with Stop solution. ODs at 405 nm without wavelength path correction were read immediately using a Varioskan LUX plate reader (Thermo Fisher Scientific Inc., Waltman, MA, USA). Curve fitting was conducted using SkanIt RE 6.1 (Thermo Fisher Scientific Inc., Waltman, MA, USA) by the linear regression of background-corrected raw OD values. Standard curve interpolated data were recorded in μmol units of catalyzed pNP per well. Alkaline activity was subsequently calculated per the equation $Activity=\left(\frac{B}{T\times V}\right)\;D$ where B is the determined umol of pNP per well, T is the reaction time, V is the sample volume, and D is the dilution factor (ng/mL). Graphical and statistical analysis of activity data were conducted in GraphPad Prism 9.0.

### 4.7. Murine Osteocalcin ELISA

Osteocalcin/Bglap (Bglap) ELISA assays (Novus Biologicals, Catalogue# NBP2-68151, Centennial, CO, USA) were performed per the vendor-supplied protocol. To measure the cell culture supernatant levels of osteocalcin, which is an indicator of osteoblastogenesis and bone matrix formation in the 3D-mcBOM. Cell cultures supernatants were collected, clarified by centrifugation (1000× *g* for 5 min), frozen, and stored at −80 °C until needed. Prior to the assay, frozen supernatants were thawed at room temperature. Samples (neat) and reconstituted standards were added to capture antibody pre-coated plates and incubated at 37 °C for 90 min. Following incubation, plates were washed 3 times with wash buffer after which reconstituted biotin-labeled antibody was added with a subsequent 60-min incubation at 37 °C. Excess biotinylated antibody solution was decanted, and plates were washed as above with the subsequent addition of streptavidin-conjugated horseradish peroxidase followed by a 30-min incubation. Following incubation, the plate was washed as above, which was followed by the addition of TMB substrate. The colorimetric reaction proceeded at 37 °C for 20 min before the addition of stop solution. OD was read at 450 nm without wavelength path correction using a Varioskan LUX plate reader (Thermo Fisher Scientific Inc., Waltman, MA, USA). Curve fitting was conducted using SkanIt RE 6.1 (Thermo Fisher Scientific Inc., Waltman, MA, USA) with a 5-parameter logistic fit model of background-corrected raw OD values to derive concentrations (ng/mL) of osteocalcin. Statistical analysis was conducted in GraphPad Prism 9.0. 

### 4.8. Alamar Blue Cell Viability Assay

Cell viability was measured in 3D-mcBOM and control samples following 35 days of culture in osteoblast differentiating or control conditions in triplicate. Alamar Blue reagent (DAL1100; Thermo Fisher Scientific Inc., Waltman, MA, USA) was diluted in complete αMEM media, which was added to wells containing 3D-mcBOM or control cultures to a final concentration of 1×. Samples were incubated at 37 °C for 3 h. Following incubation, 100 μL of media was transferred to each well of a 96-well plate. Fluorescence data were acquired using a Varioskan LUX plate reader (Thermo Fisher Scientific Inc., Waltman, MA, USA) with excitation set to 560 nm and emission set to 590 nm. Statistical analysis of relative fluorescence units (RFUs) was conducted in GraphPad Prism 9.0.

### 4.9. Histological Staining

Histological sections of 3D-mcBOM fragments were prepared from large-scale (12-well) cultures. Excised matrix fragments were fixed in 4% paraformaldehyde for 2 h and subsequently paraffin-embedded and sectioned per standard procedure (Digital Imaging and Histology Core, Rutgers-New Jersey Medical School, Newark, NJ, USA). Masson’s Trichrome staining, von Kossa staining, and Collagen 1α1 were conducted by Histowiz (Long Island City, NY, USA) per standard procedures. For cathepsin K immunostaining, the sections were deparaffinized and rehydrated prior to staining. After rehydration, the slides were soaked for 1 h in 10 mM sodium citrate (pH 6.0) at 70–80 °C for 1 h for antigen retrieval. Sections were covered in 3% hydrogen peroxide (in PBS) for 30 min and then rinsed in PBS. Sections were blocked in SuperBlock Blocking Buffer (Catalogue # PI37515, Thermo Fisher Scientific Inc., Waltman, MA, USA) at room temperature for 1 h. The sections were stained with cathepsin K primary monoclonal rabbit antibody (1:100 dilution diluted in PBS) (Novus Biologicals Lot 9H113588, Centennial, CO, USA) and incubated overnight at 4 °C. Staining was intensified by covering sections with POLINK-2 Plus Rabbit Antibody enhancer and incubating for 30 min at room temperature. Next, sections were covered with POLINK-2 Plus Rabbit polymeric HRP secondary antibody (Catalogue # D39-18 IHC World, Ellicott City, MD, USA) and incubated at room temperature for 30 min. Sections were colorimetrically developed with diaminobenzidine (DAB) (2 drops DAB chromogen/1 mL DAB substrate) (Catalogue # D39-18 IHC World, Ellicott City, MD, USA). Nuclear counterstaining was performed with Methyl green. For FITC, staining 3D-mcBOMs were stained following the methodology outlined by Ciani et al. and then imaged using a confocal microscopy (Olympus FluoView laser scanning confocal microscope) [37].

### 4.10. OsteoImage Assay

The OsteoImage assay (PA-1503; Lonza, Allendale, NJ, USA) was used to compare the hydroxyapatite content in 3D-mcBOM samples and controls as conducted per manufacturer-recommended conditions. Samples were fixed in 4% formaldehyde and 1% glutaraldehyde for ~48 h. Fixed samples were then rinsed and soaked in PBS (15 min) followed by a rinse and soak in assay wash buffer (15 min). Rinsed samples were then suspended in 1× staining reagent and were incubated at room temperature, protected from light, for 90 min. Following incubation, stained samples were washed 2 times, with assay wash buffer. Then, 2 mm sections of each sample were excised and transferred to a clear-bottom, black-walled 96-well plate and submerged in 200 μL of assay wash buffer. Fluorescence data were acquired using a Varioskan LUX plate reader (Thermo Fisher Scientific Inc., Waltman, MA with excitation set to 492 nm and emission set to 520 nm. Statistical analysis of relative fluorescence units (RFU) was conducted in GraphPad Prism 9.0. 

### 4.11. Fourier Transform-Infrared Spectroscopy

Pooled (3) 3D-mcBOM fragments were briefly fixed (4% paraformaldehyde for <2 min), washed, and subsequently stored in DI-water prior to further processing. Prior to FTIR analysis, fixed 3D-mcBOMs were dried down in an oven for 2 h at ~45 °C under slight vacuum. Dried samples were then pulverized into a fine powder via mortar and pestle. As a positive control, femoral diaphysis sections were resected from an adult C57BL/6 mouse, flash frozen, and pulverized before being oven dried and powdered as described above. FTIR transmittance spectra profiles for powdered 3D-mBOM and mouse bone samples were acquired using a Shimadzu IRSpirit Spectrophotometer (Shimadzu Scientific Instruments, Inc., Columbia, MD, USA).

### 4.12. Pierce Total Protein Assay

The Pierce Bicinchoninic (BCA) protein assay (Catalogue # 23227, Thermo Fisher Scientific, Waltman, MA, USA) was used to quantify total protein concentrations in cell culture supernatant per the manufactures recommended conditions for microplate (96-well)-based assays. Samples and media controls were diluted 20× in PBS. An albumin standard curved was prepared per the manufacturer’s protocol in PBS (2000, 1500, 1000, 750, 500, 250, 125, 50 ng/mL). Then, 25 μL of diluted sample or standard was added to 200 μL of BCA working reagent and subsequently incubated at 37 °C for 30 min. Following incubation, samples were cooled to room temperature, and OD values were read using a Varioskan LUX plate reader (Thermo Fisher Scientific Inc., Waltman, MA, USA). Protein concentration was interpolated from a standard curve fit (SkanIt RE 6.1) with a linear-regression model. Standard curve-derived concentrations were subsequently corrected for the dilution factor. Statistical analysis of protein concentration was conducted in GraphPad Prism 9.0. 

## Data Availability

The data that support the findings of this study are within this manuscript and are available by the corresponding author upon reasonable request.

## Supplementary Materials

The following supporting information can be downloaded at: https://www.mdpi.com/article/10.3390/ijms25084225/s1.
