# Supplementary material for: Methodology and Characterization of a 3D Bone Organoid Model Derived from Murine Cells
Source: Int J Mol Sci. 2024 Apr 11;25(8):4225. doi: 10.3390/ijms25084225 (PMC11050018; doi:10.3390/ijms25084225)
Supplement: Supplementary file 1 [file ijms-25-04225-s001.zip › ijms-2891240-SI.pdf]

Supplementary Figure 1. Qualitative Assessment of Relative Gene Expression from 3D-mcBOM

Relative gene expression was evaluated 3D-mcBOM for relevant osteoblastogenic and osteoclastogenic genes at different times points relative to undifferentiated control from pooled cultures at Day 35**.**

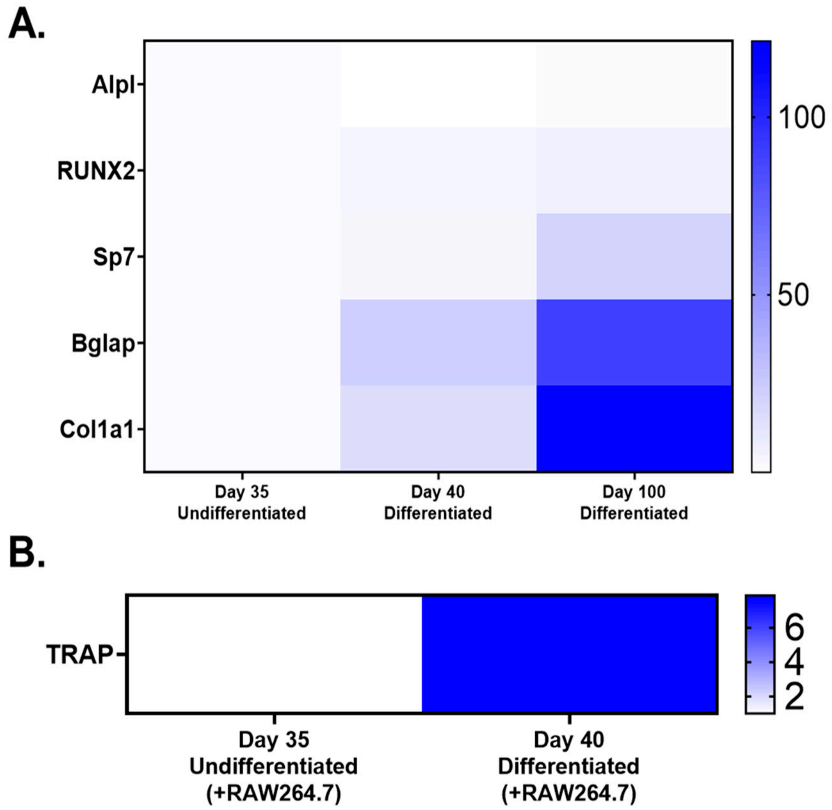

Supplementary Figure 2: Cell Viability and Protein Content

Cell viability (A) and total protein concentration (B) from 3D-mcBOM (OB+) or MC3T3-E1 cells in Matrigel (OB-) at Day 35 A pooled ratio of OB+/OB- conditions was calculated by multiplying the individual ratios for cell viability and protein concentration.

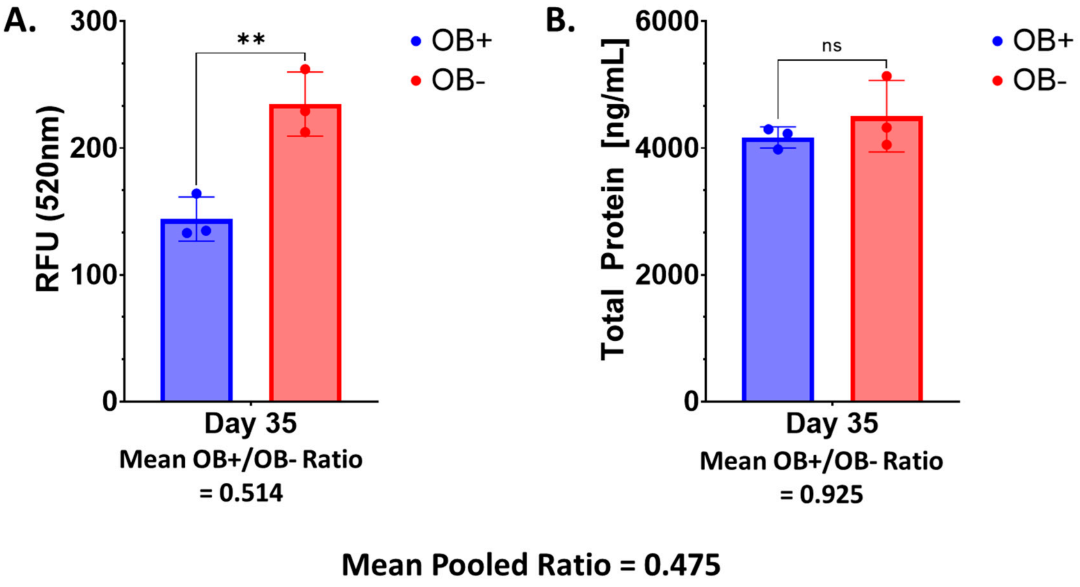

Supplementary Table 1: Summary of Experimental Outcomes for Major Components of 3D-mcBOM

Day	Process	Bone Composition & Structure	Osteoblast Function	Osteoclast Function
1-34	Osteoblastogenesis	Collagen Mineralization, Calcium Deposition (Fig3)	Hydroxyapatite quantification Osteocalcin quantification (Fig 5&8)	No Cathepsin K expression (Fig 3)
35-42	Osteoclastogenesis	Collagen Mineralization, Calcium Deposition (Fig 3)	Collagen Mineralization, Calcium Deposition, Collagen1a1 expression, ALPL analysis (Fig 3, 6-7, 9-11)	Cathepsin K expression, CTX-1 analysis (Fig 3, 6-7, 9-11)
42+	Bone Homeostasis	Collagen Mineralization, Calcium Deposition, FTIR analysis, FITC microstructure analysis (Fig 3 & 4)	Collagen Mineralization, Calcium Deposition, Col1a1 expression, ALPL analysis (Fig 3, 6-7, 9-11)	Cathepsin K expression, CTX-1 analysis (Fig 3, 6-7, 9-11)
